# Massive hemoptysis bridged with VV ECMO: A case report

**DOI:** 10.3389/fcvm.2022.997990

**Published:** 2022-09-30

**Authors:** Dylan Ryan, Kathleen Miller, Carly Capaldi, Claudine Pasquarello, Qiong Yang, Hitoshi Hirose

**Affiliations:** Department of Surgery, Virtua Health, Our Lady of Lourdes Hospital, Camden, NJ, United States

**Keywords:** ECMO, hemoptysis, ventilator, respiratory failure, ST-elevation myocardial infarction (STEMI)

## Abstract

**Objective:**

Extracorporeal membrane oxygenation (ECMO) can provide full pulmonary support when a patient is completely apneic. The combination of veno-venous (VV) ECMO and induced apnea can be utilized to control significant hemoptysis. We present a case of massive hemoptysis that developed while on VV ECMO and was treated with temporary discontinuation of the ventilator and serial declotting bronchoscopies.

**Methods:**

A 42-year-old male with recent acute ST elevation myocardial infarction status post cardiac stent developed aspiration pneumonia that progressed to acute respiratory distress syndrome. The patient's biventricular function was preserved. VV ECMO was placed for lung rescue on hospital day #7, and tracheostomy was performed for ventilator dependence on hospital day #12. On hospital day #18, the patient developed significant hemoptysis despite the discontinuation of anticoagulation. Bronchoscopy revealed massive bleeding from bilateral bronchi. To facilitate tamponade within the tracheobronchial tree, the ventilator was temporarily discontinued while VV ECMO provided full respiratory support. After 48 h, mechanical ventilation was resumed, and daily bronchoscopies were performed to remove clots from both bronchi until a chest x-ray showed improvement in bilateral opacifications. Bronchoscopy was performed a total of 14 times. There was no recurrence of bronchial bleeding, the patient's respiratory status improved, and VV ECMO was weaned off on hospital day #37. The patient was transferred to a long-term rehabilitation facility 36 days after successful VV ECMO decannulation on hospital day #73.

**Conclusions:**

This patient's survival of massive hemoptysis was facilitated largely by the utilization of serial declotting bronchoscopies with VV ECMO providing full pulmonary support during temporary discontinuation of mechanical ventilation.

## Background

The use of extracorporeal membrane oxygenation (ECMO) remains a common practice in the setting of both cardiac and respiratory emergencies (1). In patients with acute respiratory distress syndrome (ARDS), veno-venous ECMO (VV ECMO) combined with a lung-protective ventilation strategy can mitigate ventilator-associated lung injury and improve lung recovery (1, 2). In the event of substantial hemoptysis, the combination of VV ECMO and induced apnea can be utilized to control hemorrhage and allow lung healing. The following is a case of massive hemoptysis that developed while on VV ECMO and was treated with temporary discontinuation of mechanical ventilation in addition to serial declotting bronchoscopies.

## Case presentation

A 44-year-old male (weight 80 kg, height 167 cm, body surface area 1.9 m^2^) with no significant past medical history aside from vaping presented to the emergency department with anterior ST-elevation myocardial infarction (STEMI). Hypoxemia was noted with concern for aspiration and the patient was intubated. Cardiac catheterization revealed total occlusion of the proximal left anterior descending artery (LAD). An intra-aortic balloon pump (IABP) was placed, inotropic therapy was initiated, and the patient underwent successful coronary intervention of the LAD with a drug-eluting stent. Aspirin 325 mg and Clopidogrel 300 mg were introduced immediately, followed by daily dose of Aspirin 81 mg and Clopidogrel 75 mg. IABP was removed on hospital day #4 following hemodynamic improvement. Inotropic support was successfully weaned off on hospital day #6. A follow-up echocardiogram demonstrated an ejection fraction (EF) of 50% with preserved right ventricular function.

Initially, the patient maintained oxygenation with minimum ventilator support (assist control respiratory rate (RR) 16, tidal volume (TV) 500 cc, FiO_2_ 70%, PEEP 8 cm H_2_O, resulting in arterial blood gas [ABG] pH 7.32, PaCO_2_ 38 mm Hg, PaO_2_ 97 mm Hg). However, the following day, the patient's respiratory status deteriorated and his ventilator requirements quickly increased (FiO_2_ 100%, PEEP 15 cm H_2_O) after vomitus and aspiration event. On hospital day #7, the patient's hypoxia worsened (ABG: pH 7.36, PaCO_2_ 69 mm Hg, PaO_2_ 76 mm Hg) despite initiation of inhaled epoprostenol, deep sedation, paralysis, and maximum ventilator support (FiO_2_ 100%, PEEP 20 cm H_2_O, RR 26, and TV 550 cc). Repeat echocardiography showed preserved biventricular function. No inotropes or vasopressors were required to maintain adequeate hemodynamics; however, desaturation below 80% was noted by the bedside monitor. A multidisciplinary meeting was held regarding his severe hypoxia and hypercapnia, and decided to place ECMO. VV ECMO was chosen instead of venoarterial ECMO (VA ECMO) because cardiac function was preserved (2). The right femoral vein was cannulated with a 25-Fr cannula and the right internal jugular vein with a 20-Fr cannula, followed by a 5,000 unit heparin bolus (2). After VV ECMO was established, ventilator settings were adjusted to lung-protective setting (FiO_2_ 100%, PEEP 15 cm H_2_O, RR 10, TV 315 cc (ideal body weight [IBW] x 5 cc). The VV ECMO circuit was flowing 4.5–5.0 L/min with FiO_2_ 100% and sweep 8 L/min. ABG on these settings demonstrated progress with pH 7.41, PaCO_2_ 42 mm Hg, and PaO_2_ 86 mm Hg. PTT was maintained in the rage of 45–55 s with heparin per our protocol (2).

On hospital day #12 (VV ECMO day #5), tracheostomy was performed without complications. Paralytics were discontinued after tracheostomy, though lung-protective ventilator settings were maintained as FiO2 requirements began to decrease (FiO_2_ 50%, PEEP 8 cm H_2_O, RR 10, and TV 315 cc). The VV ECMO circuit was flowing nearly 5 L/min with 100% FiO2 and sweep of 9.

**Figure 1:**
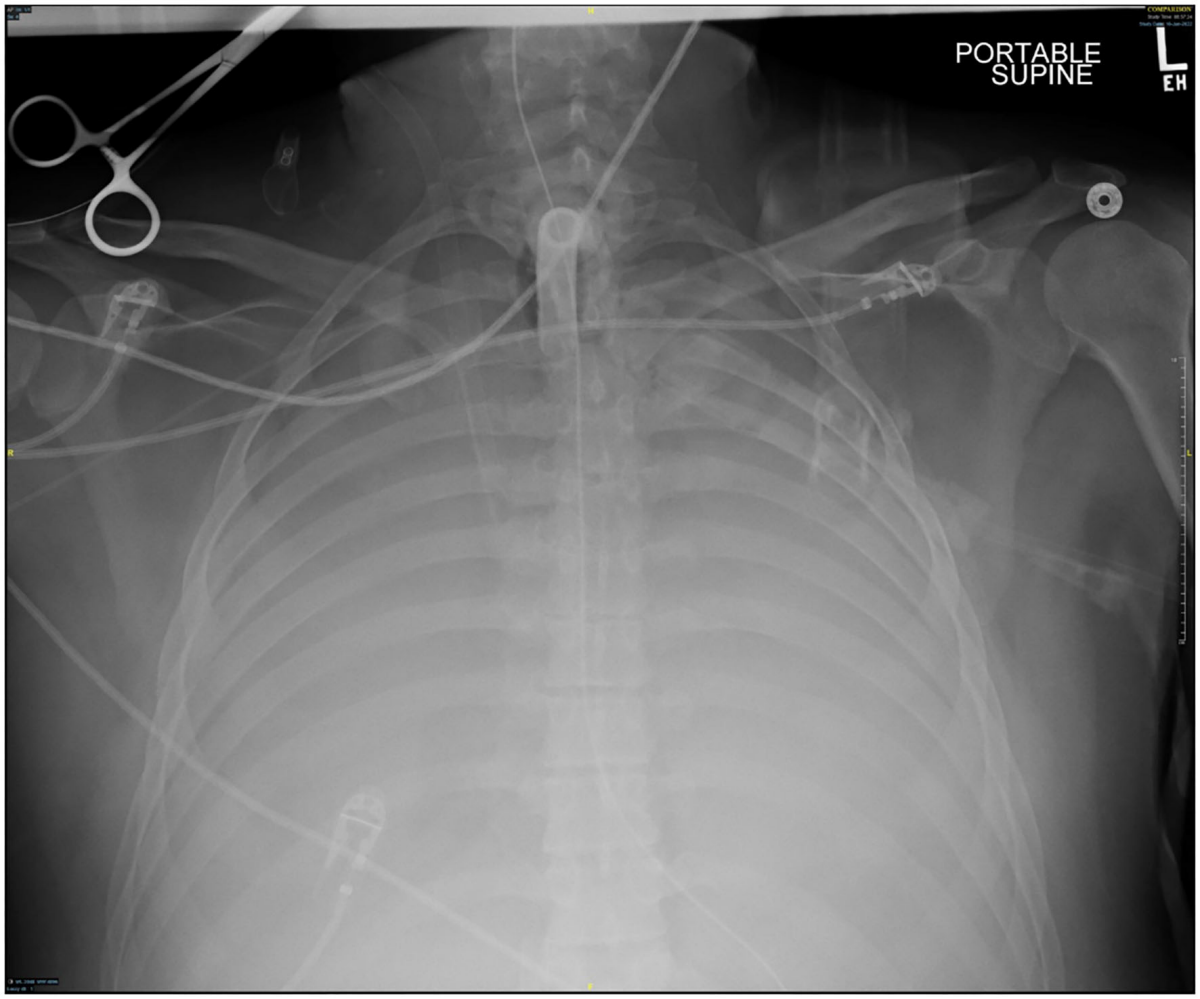
Chest x-ray at the time of hemoptysis.

**Figure 2:**
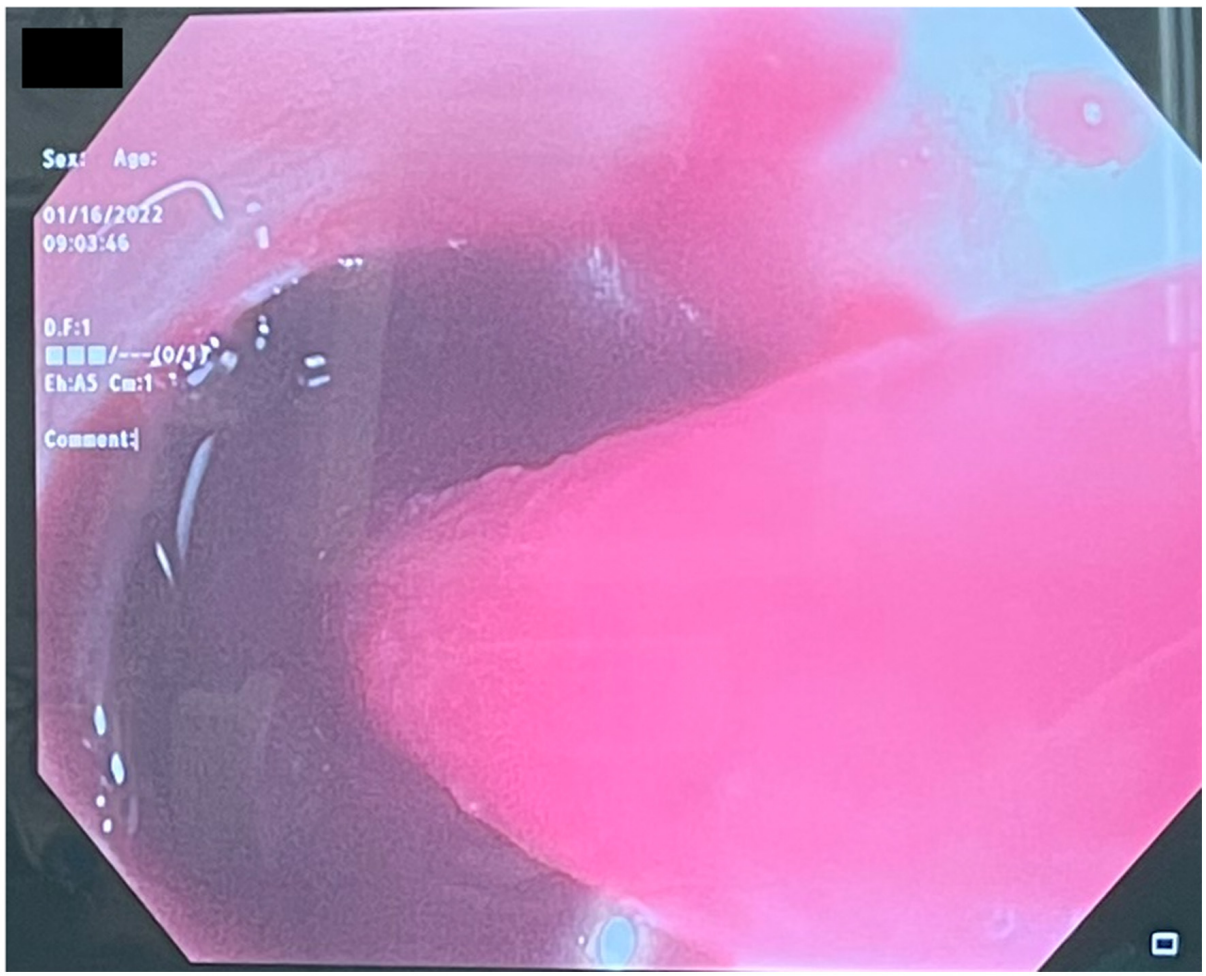
Bronchoscopy reveals massive hemoptysis.

**Figure 3:**
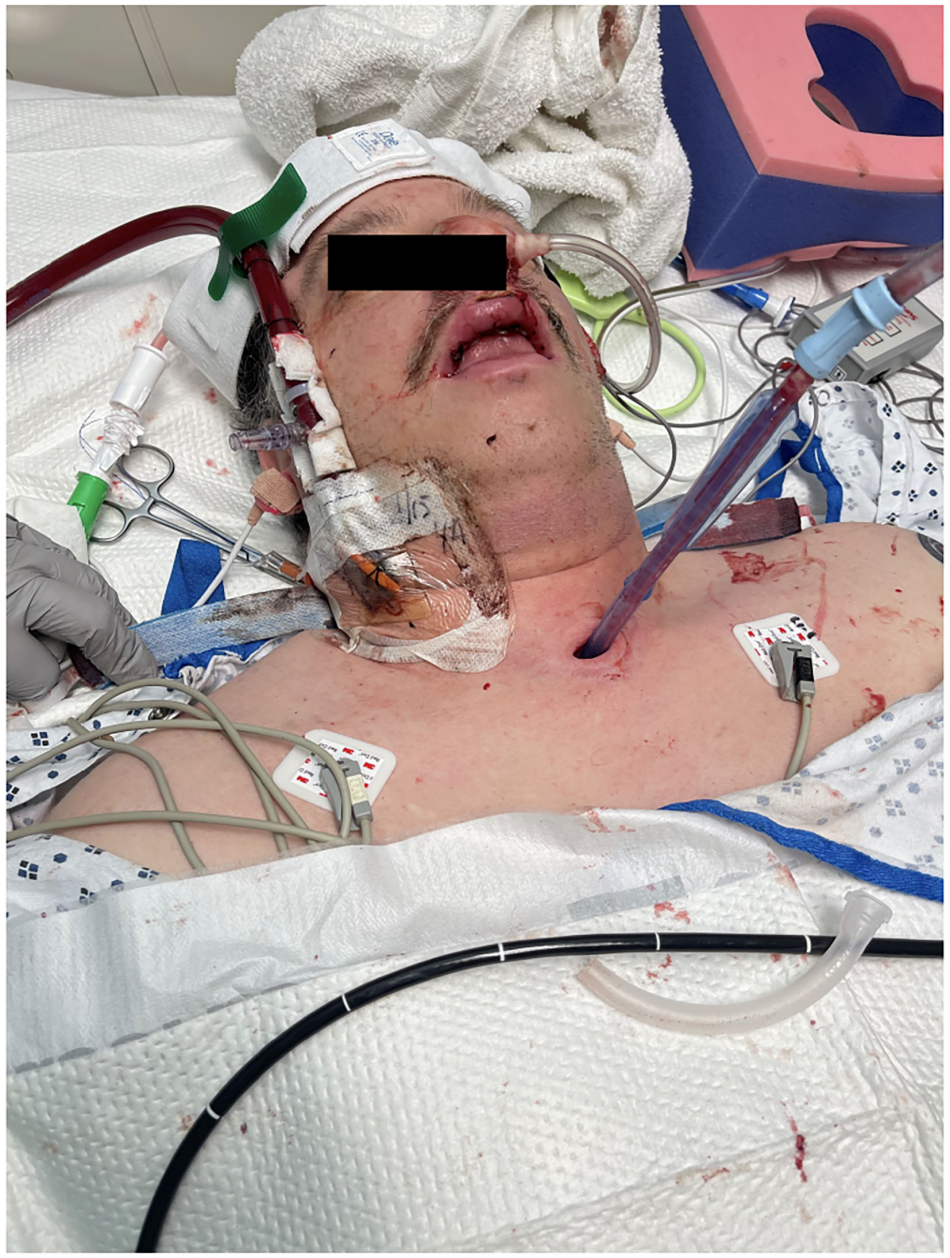
Tracheostomy tube was removed, and direct suction was performed from tracheal stoma using Yankauer catheter.

Several days after tracheostomy (hospital day #18, VV ECMO day #11) hemoptysis developed and it persisted despite holding heparin drip and normal platelet counts. His chest x-ray demonstrated bilateral opacifications (Figure 1). Dark blood was suctioned continuously from the tracheostomy and hemoglobin repeatedly dropped requiring transfusions. VV ECMO settings were increased to 100% FiO_2_ as the VV ECMO sweep was maximized to 11 L/min to maintain SpO2 >85%. Bedside bronchoscopy revealed massive bleeding in the left and right main bronchi. Ice cold saline and epinephrine lavage failed to control the bleeding, thus the site of bleeding was unable to be identified. Despite the evacuation of more than 500 cc of blood under bronchoscopy, there was continued hemorrhage from both main bronchi (Figure 2). The tracheostomy tube was removed to examine the stoma, and no active bleeding was identified in that area. A direct suction was then attempted, inserting a Yankauer suction catheter into the trachea *via* the tracheostomy stoma (Figure 3). This was also unsuccessful in clearing the airway. In further attempts to control bleeding, continuous bronchoscopy was performed for 4 h with the maintenance of adequate SpO2 >85% despite discontinuation of the ventilator.

Given adequate oxygenation, the decision was made to temporarily discontinue mechanical ventilation post-bronchoscopy to allow clot formation in the tracheobronchial tree. Paralysis was reinitiated to minimize oxygen consumption. Systemic anticoagulation and antiplatelet therapy were discontinued to promote clot formation. Due to the presence of VV ECMO, HemoSphere^TM^ (Edwards Lifesciences, Irvine, CA) technology with near-infrared tissue oximetry was utilized to sustain adequate cerebral and somatic saturation. Goals were set to maintain regional cerebral saturation >60% and somatic tissue saturation >50% for each lower extremity. Cardiac hemodynamics were evaluated non-invasively *via* FloTrac^TM^ (Edwards Lifesciences, Irvine, CA) monitoring system, with a goal cardiac index of 2.5 L/min/m^2^.

**Figure 4:**
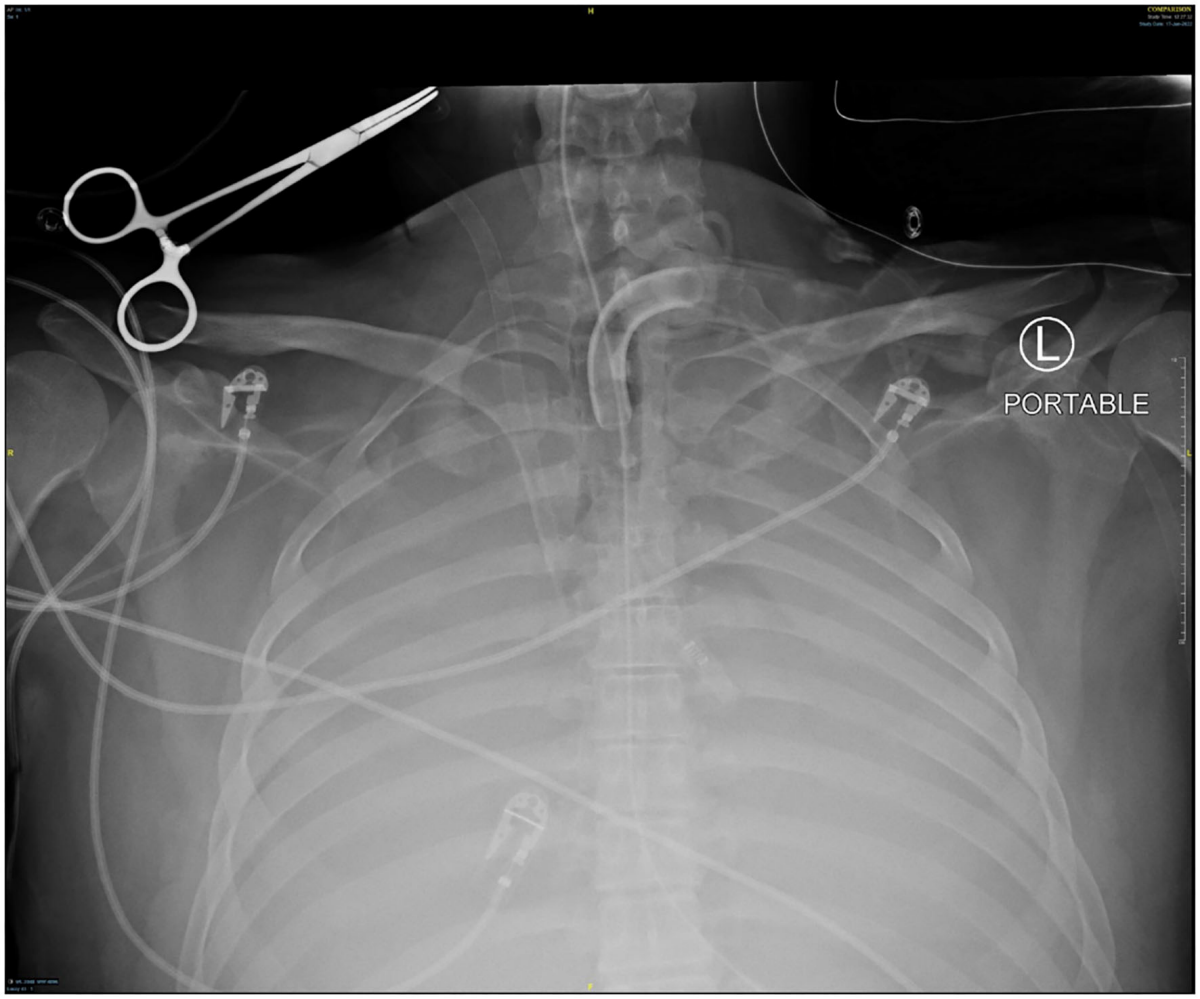
Chest x-ray off ventilator support.

**Figure 5:**
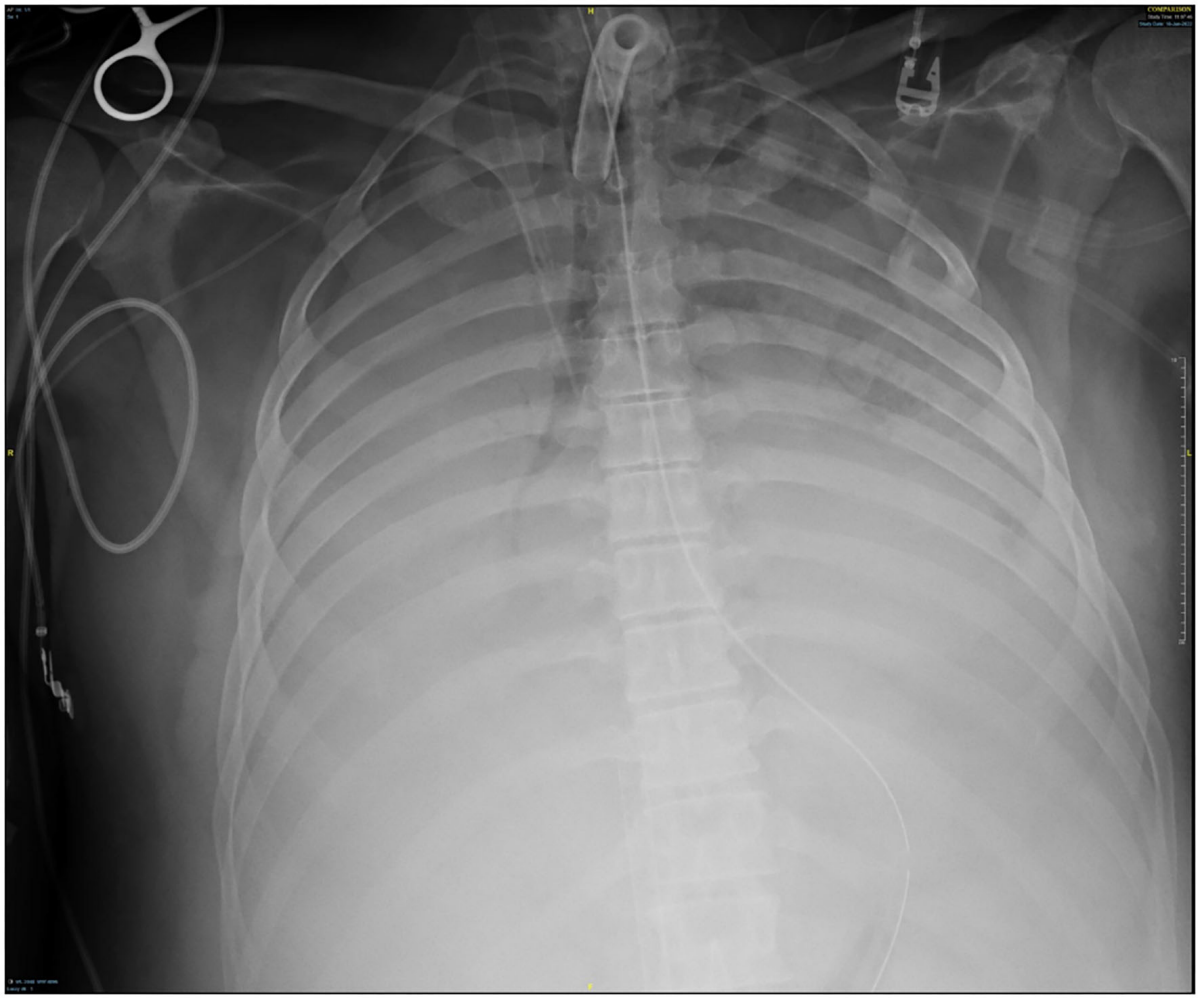
Chest x-ray 2 days after resuming ventilator.

Though active hemorrhage had ceased during this time of mechanical ventilation, the patient's chest x-ray continued to exhibit bilateral opacification (Figure 4). On hospital day #20 (VV ECMO day #13), a repeat bronchoscopy was performed to remove clot which had formed from the bilateral bronchi with some radiographic progress. Due to improvement in active hemoptysis, the ventilator was restarted with a minimum TV of 250 cc (IBW x 4 cc) after 48 hours of complete discontinuation of ventilator. Serial declotting bronchoscopies were then performed utilizing various bronchoscopic instruments including a basket, biopsy clamp, and brush to facilitate clot retrieval. This process was continued daily until all visible clot was removed and chest x-ray demonstrated decreased opacification (Figure 5). Bronchoscopy was performed a total of 14 times. Antiplatelet therapy was then resumed, and there was no recurrence of bronchial hemorrhage, although systemic heparin and anti-platelet agents were held.

**Figure 6:**
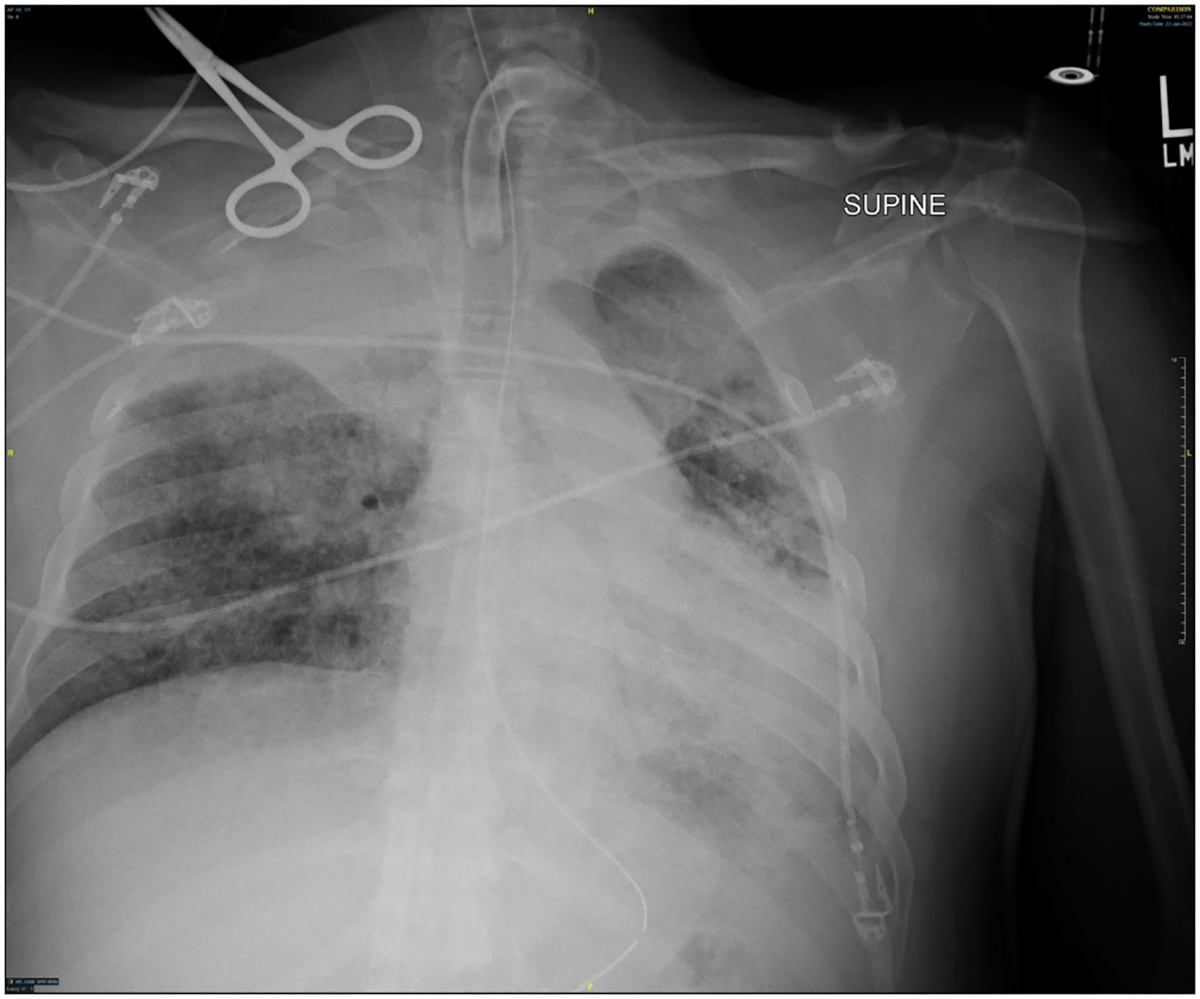
Chest x-ray 4 days after resuming ventilator.

Despite low tidal volumes, the patient's peak airway pressure remained elevated above 40 cm H_2_O. Due to rising peak airway pressures, the ventilator mode was switched to pressure control ventilation (PCV) with 15 cm H_2_O above PEEP 15 cm H_2_O. Over time, chest x-ray findings improved on these ventilator settings, with TV ~50cc (Figure 6). Sweep gas was adjusted to meet a goal PaCO_2_ ~40 mm Hg, and TV was gradually increased. Once an appropriate TV of 300 cc was consistently achieved by PCV, paralysis was discontinued. At that time, the ventilator mode was changed from PCV to Volume Control (VC) with a tidal volume of 315 cc (IBW x 5cc).

Of note, the patient's hospital course was further complicated by acute renal failure requiring continuous veno-venous hemodialysis (CVVHD) and *Serratia* and *Klebsiella pneumonia* treated with the appropriate antibiotics.

**Figure 7:**
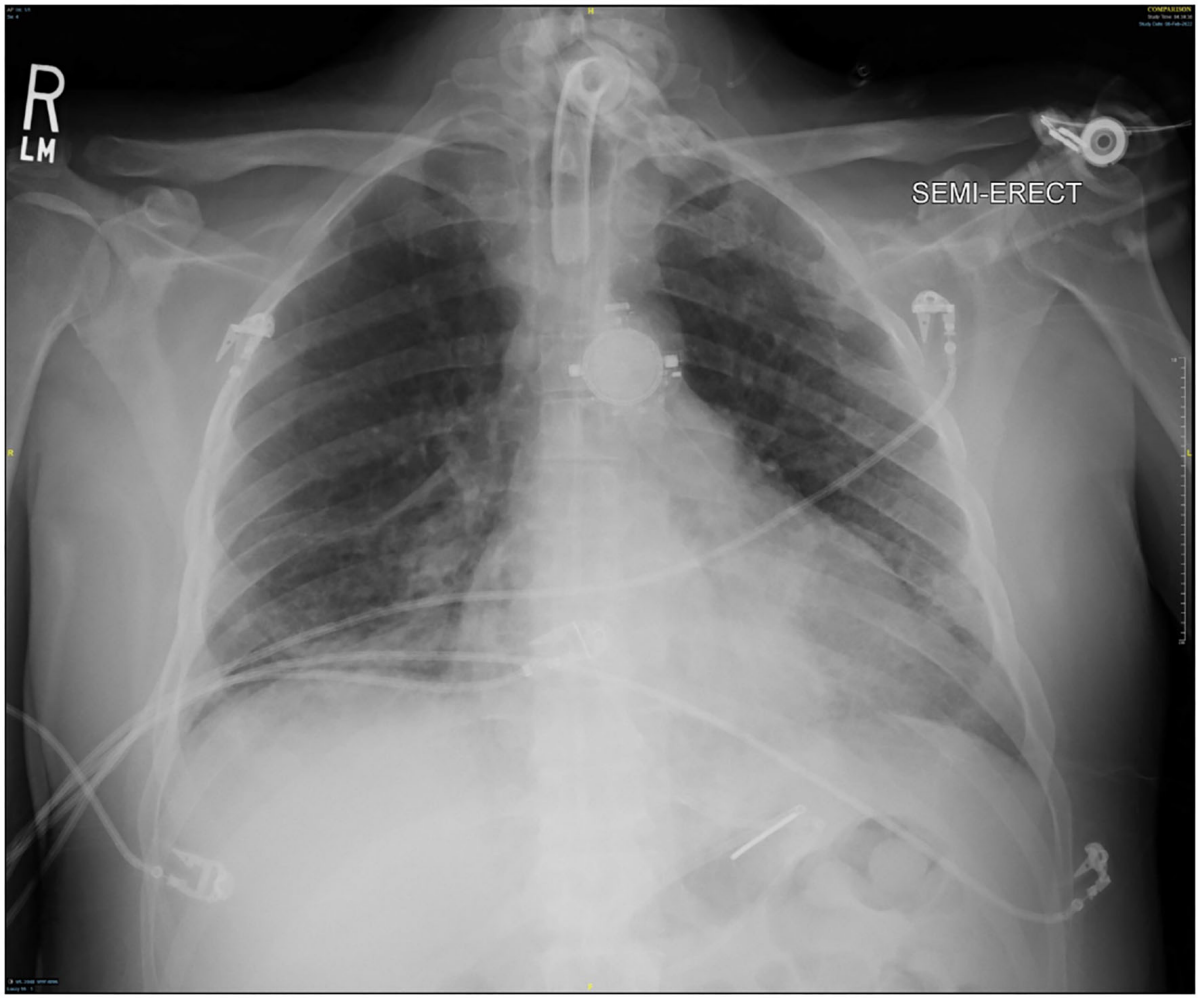
Chest x-ray after ECMO decannulation.

As his respiratory status improved, VV ECMO FiO_2_ and sweep were decreased as tolerated. VV ECMO sweep gas was eventually minimized and his ABG remained appropriate with standard ventilator support of 50% FiO_2_, PEEP 10 cm H_2_O, and TV 570 cc. With significant radiographic improvement (Figure 7), VV ECMO was successfully decannulated at the bedside on hospital day #37 (VV ECMO day #30). There was no thrombotic complication or pump thrombosis even though we held anticoagulation while VV ECMO run. CVVHD was converted to hemodialysis on hospital day #42 (VV ECMO removal day #5). The ventilator was eventually switched to pressure support ventilation (PSV) which the patient tolerated well. The patient was mobilized to the chair daily and was able to communicate without evidence of neurological compromise. On hospital day #73 (VV ECMO removal day #36), the patient was successfully discharged to an acute rehabilitation facility. The patient had an outpatient office visit 3 months after VV ECMO discontinuation without the need of tracheostomy or hemodialysis. At that time, his oxygen saturation was 98% with 2L nasal cannula supplemental oxygen and he was able to walk without assistance. There was no desaturation with 6 min walk test. He was eventually return to work without physical disability.

## Discussion

In the setting of massive hemoptysis, VV ECMO has been used for rescue oxygenation and ventilation support (3–5). In prior cases, ECMO was used as a bridge to definitive treatment but was not utilized in conjunction with prolonged induced apnea. A contributing author in this case study has previously reported VA ECMO support for massive hemoptysis (6). The case study reinforced discontinuation of mechanical ventilation under ECMO support, but was used, in conjunction with bronchial artery embolization by interventional radiology and subsequent serial bronchoscopies for clot removal.

Induced apnea *via* temporary ventilator cessation can be safely used to facilitate airway clot formation while using VV ECMO as primary support for adequate oxygenation and ventilation. This strategy can lead to eventual tamponade of bleeding sites. Alternatively, if the active bleeding can be localized to a single site, a bronchial blocker and one lung ventilation approach may be utilized (7). Bronchial blocker with one lung ventilation was not feasible in the case presented due to diffuse bilateral bleeding. Additionally, the use of bronchial artery embolization was not possible as with bilateral bleeding there was likely a need for multiple embolizations of the bronchial and/or pulmonary arteries.

Although ECMO provided sufficient oxygenation during ventilator cessation, systemic circulation was monitored by conventional pulse oximetry, arterial blood gases, cerebral/somatic tissue saturation, and cardiac output provided *via* FloTrac^TM^. Near-infrared technology was essential to ensure appropriate cerebral oxygenation during deep sedation (5). With continuous cerebral saturation data, sedation was adjusted confidently to maintain adequate oxygenation and allow resting of brain activity (8).

While on VV ECMO, appropriate cardiac output must be maintained in order to circulate oxygenated blood from the right side of the heart *via* native cardiac ejection (9). This requires preserved cardiac function. If cardiac status is depressed, VA ECMO would be a more appropriate modaility for mechanical circulatory support, rather than VV ECMO. In this case, VV ECMO was selected because patient had preserved biventricular function.

Monitoring cardiac function in patients with massive hemoptysis is essential as some may develop cardiac dysfunction either from hypoxia or stress-induced cardiomyopathy. This was assessed intermittently *via* transthoracic echocardiography and continuously *via* FloTrac^TM^ technology throughout the patient's hospital course. New technology provided by FloTrac^TM^ uses an arterial pressure waveform to calculate left-sided cardiac output using the patient's arterial line (10). Non-invasive hemodynamic monitoring is preferred since thermodilution cardiac output assessments are not accurate in patients on VV ECMO due to the return of blood from the ECMO circuit to the right side of the heart. Mixed venous saturations are also falsely elevated for this reason. In this case, the non-invasive FloTrac^TM^ device was primarily utilized while on VV ECMO to ensure that sufficient cardiac output was maintained.

The etiology of massive hemoptysis in this patient remains unknown (7). The initial presumed cause was trauma to the tracheobronchial tree during tracheostomy; however, the tracheostomy stoma was without hemorrhage on multiple inspections. Additionally, the carina was evaluated and no injury was localized. Anticoagulation-induced bleeding is common in ECMO patients; however, it is less likely the source in this study as PTT was tightly controlled within the 45–55 range as per our protocol (11). Another etiology considered was medication induced platelet dysfunction given recent cardiac stenting after anterior STEMI. This was later deemed unlikely as bleeding did not recur upon reinitiation of antiplatelet therapy. Hemoptysis secondary to tracheo-innominate artery fistula is typically seen as a late complication following tracheostomy but has been reported to develop as early as 3 days post procedure (12). Bleeding from this type of fistula is classically seen to the right of the 6–10th tracheal rings, located in close proximity to the innominate artery (13). Mortality from tracheo-innominate fistula without surgical or endovascular intervention is reported to be nearly 100% (13). In this case, bleeding ceased without surgical or endovascular involvement, suggesting the source of hemorrhage was more likely in the bilateral lower airways than from a tracheo-innominate fistula. Lastly, diffuse alveolar hemorrhage was considered. It can be seen in severe inflammatory lung disease and is diagnosed *via* lung biopsy. This cause cannot be confirmed here as a lung biopsy was not obtained; however, diffuse alveolar hemorrhage was ultimately suspected as the etiology of bleeding in this case.

We propose the following clinical pathway for a patient with massive hemoptysis: (1) appropriate selection of ECMO support, (2) discontinuation of anticoagulation and antiplatelet therapy, (3) bronchoscopy to identify bleeding sites, (4) bronchial blocker or one lung ventilation if bleeding is localized, (5) induced apnea with mechanical ventilation cessation and full respiratory support *via* VV ECMO to facilitate clot formation, (6) initiation of deep sedation and paralysis, (7) continuous cardiac output and cerebral saturation monitoring, (8) serial bronchoscopies for clot removal following successful tamponade *via* the above measures, and (9) progressive return to ventilator support.

## Conclusion

Massive hemoptysis while on ECMO can be managed with induced apnea by temporarily discontinuing the ventilator and utilizing VV ECMO as primary support for oxygenation and ventilation. This combination is best used to provide full pulmonary support and safely allows tamponade of bleeding sites. Following hemorrhage cessation, serial bronchoscopies can be performed to remove formed clots from the airways. With time, mechanical ventilation can be gradually resumed and VV ECMO support decreased. In successful cases, there will be a reduction in oxygen requirements and radiographic improvement of the lungs with the resolution of lung opacification. Because of this, mechanical pulmonary support with VV ECMO can be successfully weaned while maintaining satisfactory gas exchange as hemoptysis resolves.

## Data availability statement

The raw data supporting the conclusions of this article will be made available by the authors, without undue reservation.

## Ethics statement

The studies involving human participants were reviewed and approved by Virtua Health. The patients/participants provided their written informed consent to participate in this study. Written informed consent was obtained from the patient for the publication of any potentially identifiable images or data included in this article.

## Author contributions

All authors listed have made a substantial, direct, and intellectual contribution to the work and approved it for publication.

## Conflict of interest

Author DR serves as a clinical consultant of Edwards Lifesciences. The remaining authors declare that the research was conducted in the absence of any commercial or financial relationships that could be construed as a potential conflict of interest.

## Publisher's note

All claims expressed in this article are solely those of the authors and do not necessarily represent those of their affiliated organizations, or those of the publisher, the editors and the reviewers. Any product that may be evaluated in this article, or claim that may be made by its manufacturer, is not guaranteed or endorsed by the publisher.
